# Raman Spectroscopy as a Robust New Tool for Rapid and Accurate Evaluation of Drought Tolerance Levels in Both Genetically Diverse and Near-Isogenic Maize Lines

**DOI:** 10.3389/fpls.2021.621711

**Published:** 2021-07-12

**Authors:** Narangerel Altangerel, Pei-Cheng Huang, Michael V. Kolomiets, Marlan O. Scully, Philip R. Hemmer

**Affiliations:** ^1^Institute of Quantum Science and Engineering, Texas A&M University, College Station, TX, United States; ^2^Physics Department, Baylor University, Waco, TX, United States; ^3^Plant Pathology and Microbiology Department, Texas A&M University, College Station, TX, United States; ^4^Mechanical and Aerospace Engineering, Princeton University, Princeton, NJ, United States; ^5^Electrical and Computer Engineering Department, Texas A&M University, College Station, TX, United States

**Keywords:** lipoxygenase, Raman spectroscopy, carotenoid degradation, osmotic stress, drought stress, maize inbred lines

## Abstract

Improving drought tolerance of crops has become crucial due to the current scenario of rapid climate change. In particular, development of new maize germplasm with increased drought tolerance is viewed as a major breeding goal to ensure sustainable food and feed production. Therefore, accurate rapid phenotyping techniques for selection of superior maize genotypes are required. The objectives of this study were to determine whether Raman microscopy technique can be applied for accurate assessment of drought-tolerance levels in both genetically diverse and near-isogenic maize lines that differ in their levels of drought-tolerance. Carotenoid degradation is known to be a direct stress response initiated by reactive oxygen species during osmotic stress such as drought. Using Raman mapping, we observed real-time changes in the rate of carotenoid degradation in chloroplasts that was dependent on the strength of osmotic stress. In addition, we showed that the rate of carotenoid degradation as measured by Raman spectroscopy correlates directly with drought tolerance levels of diverse maize genotypes. We conclude that Raman technique is a robust, biochemically selective and non-invasive phenotyping technique that accurately distinguishes drought tolerance levels in both genetically diverse and near-isogenic maize genotypes. We conclude that this technique can be further developed to render it suitable for field-based early assessment of breeding materials with superior drought-tolerance traits.

## Introduction

Due to rapidly increasing human population, there is a crucial need to meet global food security needs and satisfy the increasing demand for food, feed, and fuel. To do so, it is necessary to provide improved plant phenotyping techniques to breeding programs to aid in the development of germplasm of crops that have increased drought tolerance levels, and can thus help adapt to future climate changes (Borlaug, 2000; De La Fuente et al., 2014). Improving drought tolerance of crops has become one of the significant challenges in global agricultural production and food security (Tilman et al., 2001; Chaves et al., 2003; De La Fuente et al., 2014).

Maize (*Zea mays*) is the world's most important production crop: its starch, protein, and oil are essential for supplying adequate food and nutrition to both human and animals (Ranum et al., 2014). In addition, maize starch has recently become an important feedstock for ethanol production (Ranum et al., 2014). A major objective in most breeding programs is to develop superior germplasm that tolerate water stress yet produce a higher yield. This is achieved by employing vastly improved breeding strategies through years of genetic research, including marker assisted selection and hybrid seed production (Fiorani and Schurr, 2013). The phenotype of a plant is defined by both differences in genome and their interactions with the environment. Therefore, genotypes that correlate with improved yield or other agronomic traits must be considered in the contexts of various environments to determine optimum phenotypic responses (Fiorani and Schurr, 2013).

The majority of phenotypic traits of genotypes consist of various morphological, physiological, and biotic stress traits; however, the current breeding program also includes the evaluation of germplasm performance under drought conditions. One of the major drawbacks of conventional phenotyping techniques is the long period of time it takes to measure the effect of stress on plants. Current *in vivo* spectroscopic techniques such as reflectance spectroscopy (Gutelson and Merziyak, 1996), IR thermal imaging (Zia et al., 2013), hyperspectral imaging (Behmann et al., 2014), and chlorophyll fluorescence spectroscopy (Kalaji et al., 2011) are limited by the time required for sensing a drought stress response and the level of the stress. Chemical extraction techniques are more relevant but are destructive and labor-intensive.

Because of unpredictable weather patterns, drought tolerance screening of breeding populations during the entire growing session over many months is difficult to perform as drought stress is difficult to control. To speed up this research, there is an urgent need to develop more robust phenotyping techniques for non-destructive, accurate and rapid assessment of breeding populations for drought related responses, especially at early seedling stages and with short periods of withholding water.

Here, we investigated whether Raman spectroscopic techniques can overcome these limitations of traditional phenotyping by achieving the necessary biochemical sensitivity *in vivo*. For this demonstration, we chose to analyze drought stress tolerance among 5 genotypes of maize. Specifically, we measured drought tolerance levels by assessing the level of transpirational water loss among the young seedlings during a week in which water was withheld. Measuring drought tolerance in seedlings is of interest because it shortens the time needed to evaluate different genotypes. Previously, we demonstrated the use of Raman spectroscopy for early *in vivo* detection of plant stress in mature plants, where we found that plants exposed to multiple abiotic stresses such as cold, excess light, saline and drought stress conditions display rapid degradation of carotenoids and accumulation of anthocyanins (Altangerel et al., 2017a). In that study, we used 5 months old genetically identical *Coleus* plants belonging to a single variety. Here, we used two-week old seedlings of genetically diverse maize and near-isogenic inbred lines that differ in their responses to drought stress in an attempt to evaluate whether differences in phenotypic responses to drought stress can be accurately assessed by Raman spectroscopy. Because young seedlings produce anthocyanins for purposes unrelated to drought stress (Karageorgou and Manetas, 2006), we have decided not to assess the level of anthocyanins to study stress in these plants. Instead, we monitored the rate of carotenoid degradation during drought stress for each genotype.

Plants undergo highly intricate physiological, biochemical, and molecular changes when they are exposed to drought (De La Fuente et al., 2014). Notably, highly toxic reactive oxygen species (ROS) accumulate in plants during drought conditions. Carotenoids play irreplaceable roles during abiotic stress such as drought by quenching chlorophyll-excited states, scavenging ROS, and dissipating excess energy to heat (Krinsky, 1989; Muller et al., 2001; Davison et al., 2002; Krieger-Liszkay et al., 2008; Shan and Li, 2008; Yoshikazu et al., 2008; Ramel et al., 2012). They are considered to be the first line of defense against ROS, serving as the main ^1^O_2_ quencher in chloroplasts (Krinsky, 1989; Davison et al., 2002; Shan and Li, 2008; Ramel et al., 2012). The oxidative degradation of carotenoids, like the accessory photosynthetic pigment β-carotene, causes accretion of several volatile β-carotene derivatives such as β-cyclocitral. Beta-cyclocitral is a molecular signal responsible for induction of ^1^O_2_-responsive genes (Davison et al., 2002; Ramel et al., 2012). Therefore, rapid conversion of β-carotene to β-cyclocitral during oxidative stress is an important line of defense against osmotic stress (Davison et al., 2002; Ramel et al., 2012).

At the same time, β-carotene, along with other carotenoids, absorbs well in the high energy blue spectrum of visible light and are used as accessory pigments in light harvesting complexes (Krieger-Liszkay et al., 2008; Yoshikazu et al., 2008). These are not core elements of the light-harvesting complex but instead are used to saturate energy transfer into the reaction center after excitation at wavelengths varying from those of the more abundant chlorophyll. Degradation of carotenoids reduces saturation, protecting the photosystem core pigments in the reaction center and membrane tissues from the effects of photooxidation (Muller et al., 2001).

Here we demonstrated real-time carotenoid degradation in chloroplasts during drought stress using the combination of our newly developed immediate osmotic stress technique and Raman mapping. Then, we demonstrated Raman technique's ability to sense different rates of carotenoid degradation under various strengths of osmotic stress. At last, we showed that the degradation rate of the carotenoids, as measured by the Raman microscopy technique, can be used to accurately distinguish drought tolerance levels in both genetically diverse and near-isogenic maize genotypes within a short period from several days to a week of withholding water.

## Materials and Methods

### Plant Material and Growth Conditions

Three maize inbred lines differing in their levels of drought responses, B73, CML176, and OH28, were used in this study. In addition, lipoxygenase mutants with contrasting drought tolerance phenotypes, *lox2* and *lox4* mutants, were back-crossed seven times to the recurrent parent B73 to generate near-isogenic mutants which share ~99.3% genome identity (Huang, 2017). Maize seedlings were grown in conical pots (15 by 3 cm) in the same amount (dry weight) of sieved sterile (autoclaved) commercial potting mix (Metro-Mix 366; Scotts-Sierra Horticultural products, Marysville, OH, U. S. A.) at 22°C under a 14-h day length, 50% humidity, 200 μmol m^2^/s of light in the laboratory.

Specifically, two replications of six B73 maize plants per replication were used for the *in-situ* Raman study of immediate osmotic stress in plant tissues. We grew these plants under two different lighting conditions such as normal and completely dark conditions. The normal lighting condition was a 14-h length of 200 μmol m^2^/ s of light and a 10-h length of darkness. The completely dark condition was 24-h darkness for a day.

Three of the plants in each replication grew under normal growth conditions and had normal photosynthesis activities and chlorophyll contents. The other three plants in each replication grew under complete darkness which resulted in lower photosynthetic activities due to extremely low content of chlorophyll.

Three replications of 10 plants per replication per genotype were used for the *in-vivo* Raman study. Before withholding water, all experimental plants were soaked in deionized water until reaching 100% water content in their soil. Then, five plants of each genotype were well watered every day and served as a control group in this study. The other five plants of each genotypes were kept withholding water for a week, and served as drought stressed group in this study. After a week of *in-vivo* Raman measurements, all experimental plants were harvested, and root and shoot tissues were separated for dry weight measurement.

### Transpirational Water Loss Measurement

Transpirational water loss measurements were performed with slight modifications following the protocol from (Ruggiero et al., 2004). Fourteen days after sowing, pots with seedlings which have two leaves with visible leaf collars (V2 stage) were soaked in deionized water for more than 30 min to reach 100% water content in the soil before withholding water. After the seedlings were soaked in deionized water, each pot was covered with a para-film to avoid water loss from soil surface. Three plants were then placed into a bigger pot as one sample and the pot weight was measured every day and the difference of pot weight represents the amount of water lost via transpiration. Water loss through transpiration was normalized by plant dry weight taken at the end of the experiment.

### *In situ* Raman Studies*:* Simulating Immediate Osmotic Stress in Plant Tissue and Raman Microscopic Mapping

**Figure 1:**
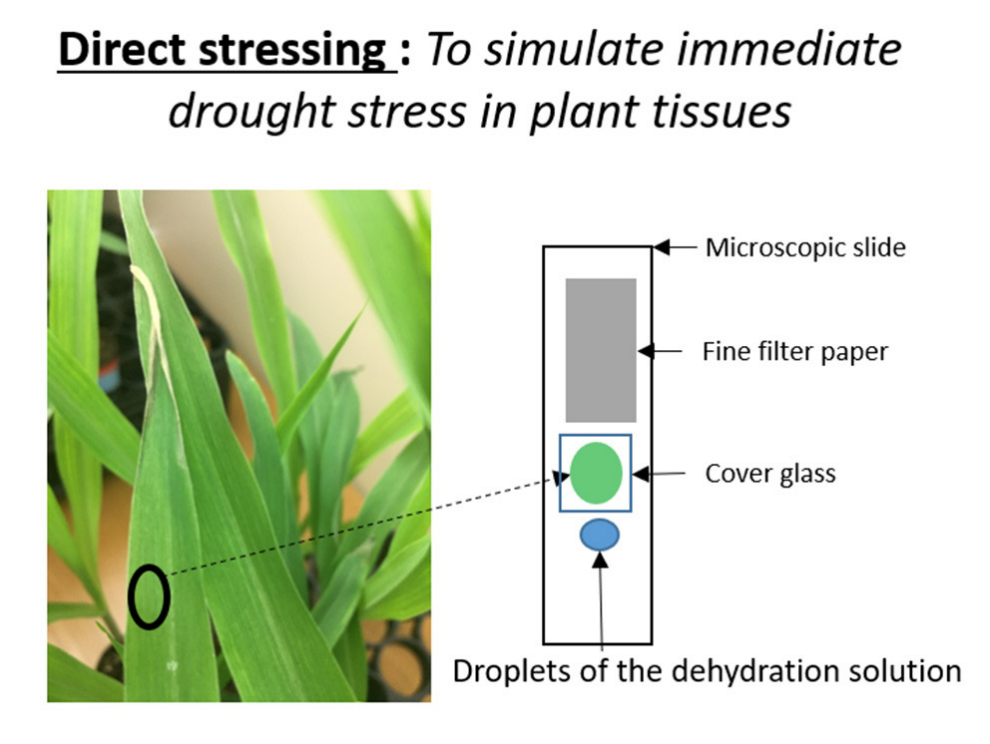
Illustration of the immediate osmotic stress technique.

To simulate immediate drought stress in leaf tissue, we developed a rapid drought stressing technique that uses a dehydrating chemical, mannitol. Watering plants with mannitol solutions chemically induces osmotic stress (Fritz and Ehwald, 2011). Osmotic stress occurs in plants during drought or high salinity conditions. First, we cut about a 1 cm^2^ leaf disk from our experimental subjects of two-week old B73 plants. We then wet-mounted it on a microscopic slide in a manner that allowed the leaf tissue to be suspended inside deionized water between the microscopic slide and cover glass. We introduced the dehydration solution to the plant tissue using the capillary effect (Figure 1). In order to do so, we dropped 2 drops of mannitol solution near the left edge of the cover glass, holding a filter paper (Whatman, filter 1) near the right edge of the cover glass. Once the solution started to enter between the cover and microscope glasses, the same amount of water was absorbed by the filter paper. Meanwhile, we made sure the location and height of the Raman sampling area stayed the same during this process by observing it with a camera. When mannitol solution enters the tissue area, it creates immediate osmotic stress on the tissue. Here, we used 100, 150, and 250 mM of mannitol solutions to imitate different strengths of osmotic stresses. We took Raman maps before and after introducing mannitol solutions to the wet-mounted plant tissues. Raman mapping was done by a Raman confocal microscope system with an excitation laser of 532 nm laser. The laser sampling size was 2 microns with 2 mW power and 0.5 s acquisition time. Each XY map size was 180 by 140 microns with 80 sampling points, and each XZ map was 160 microns by 15 microns with 30 sampling points. All Raman images are based on the heights of carotenoids' 1,157 cm^−1^ peak.

### *In vivo* Raman Study: Raman Microscopic Measurements, Data Processing, and Statistical Analysis

We used a Raman confocal microscopic system with a 532 nm CW laser for microscopic measurements (Horiba, LabRam HR Revolution), placing plant leaves directly onto the sample holder without physical detachment from the plant. Thus, we used *in vivo*, or non-invasive detection. Air-cooled CCD cameras efficiently detected the laser induced scattered radiation (signal). The laser powers were adjusted to be low enough to not affect the live cells in plant tissue. The laser sampling spot size was 2 microns, and the laser power was 0.5 mW with 1 second acquisition time for microscopic measurements. Twelve Raman spectra were collected from 3rd and 4th leaves of each plant because 1st and 2nd leaves had signs of senescence on drought stressed plants. We took the Raman spectral data of the plants (leaves) every 24 h for 7 days between 8 a.m. and 12 p.m. during the on-set and development of stress. Because plant leaf is a complex system, we used the mean spectra, averaged over different regions, for further analysis. Since the greatest contributor of noise to Raman spectra is the intrinsic fluorescence of molecules in plant tissues, we removed the fluorescence background in order to extract accurate Raman signals from the raw spectra. The baselines of Raman raw spectral data were corrected by fitting the background with high order polynomials with multiple iterations (Lieber and Mahadevan-Jansen, 2003). The spectra were then smoothed by the Savitzky-Golay algorithm with 15 adjacent points and normalized by the unit vector method, a common normalization method for Raman spectra of biological samples. All data processing programs were written in MATLAB R2013a (The Mathworks, Natick, MA, USA). Results are reported as means ±SE, from 3 different experiments. Data were evaluated using one-way analysis (ANOVA) using the Origin Labs 8.1 and *P* < 0.05 were considered to indicate statistical significance.

## Results and Discussion

### *In situ* Raman Studies: Real-Time Carotenoid Degradations During Osmotic Stress

Since the major objective of this study was to determine whether Raman spectroscopy can be utilized to distinguish maize genotypes that display diverse levels of drought tolerance, we first aimed to establish whether carotenoid degradation is proportional to the levels of osmotic stress that leaf tissue experiences by using different concentrations of mannitol treatments of leaf disks. Carotenoids are yellow to orange accessory photosynthetic pigments that have nutritional relevance as an antioxidant and are well-studied for their relevance to plant stress responses (Krinsky, 1989; Muller et al., 2001; Davison et al., 2002; Krieger-Liszkay et al., 2008; Shan and Li, 2008; Yoshikazu et al., 2008; Ramel et al., 2012). Degradation of carotenoids is one of the plant's direct responses to stress, and in our previous study, using Raman microscopy, we observed this degradation in response to multiple stress conditions in genetically identical *Coleus* plants (Altangerel et al., 2017a).

**Figure 2:**
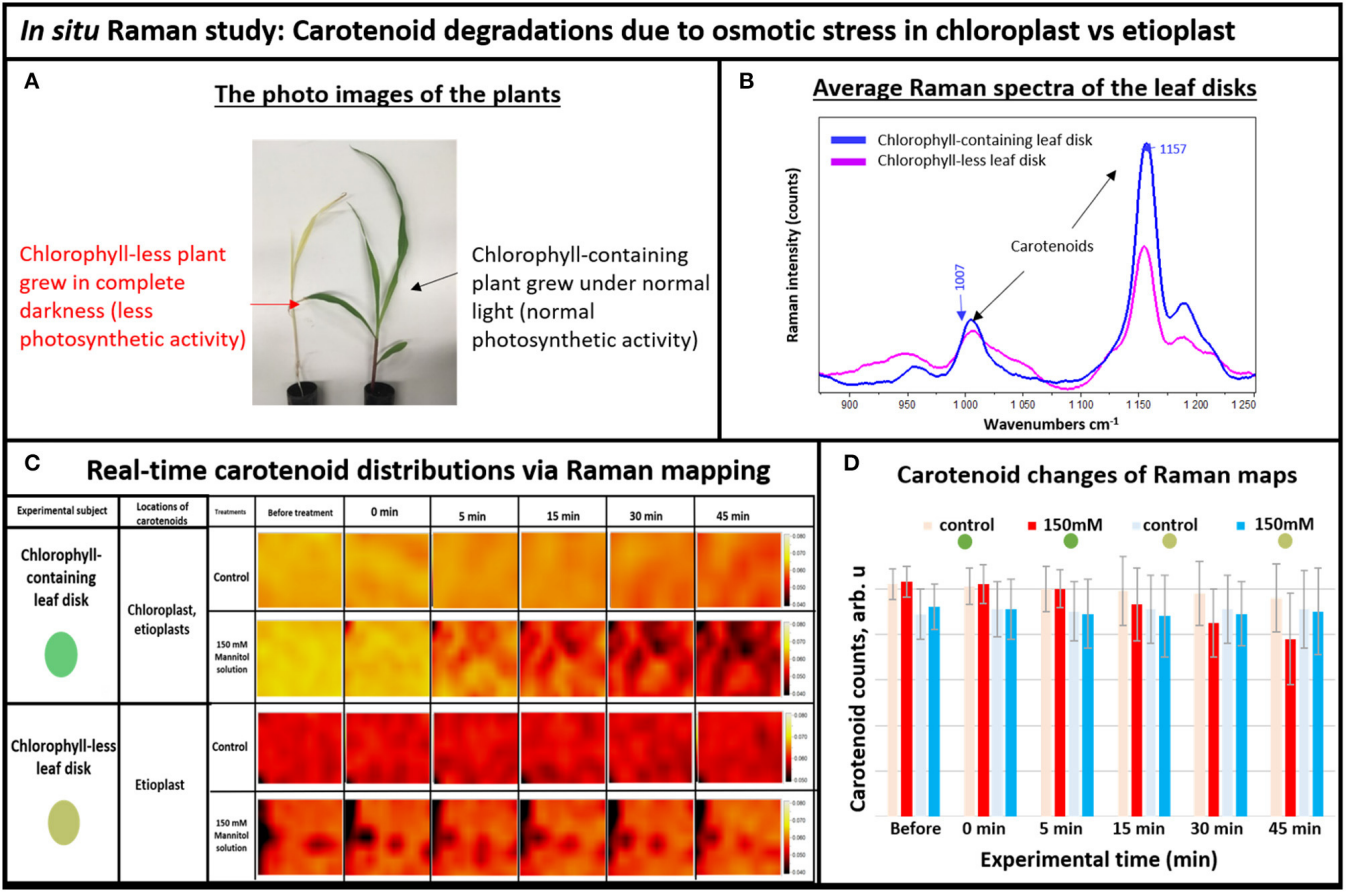
**(A)** The photo images of the experimental plants: chlorophyll-containing (green) vs. chlorophyll-less (white), **(B)** Average Raman spectra of the chlorophyll-containing and chlorophyll-less leaf disks, **(C)** Real-time carotenoid distribution changes due to the 150 mM mannitol solution vs. pure deionized water, green and white leaf disks (areas of 170 × 140 μm): before treatments and 0, 5, 15, 30, and 45 min after the treatments. **(D)** Histogram of carotenoid changes of Raman maps. Each bar represents mean +SD (*n* = 80 × 3 × 2).

In plants, degradation of carotenoids happens slowly, within days. We reasoned that if degradation of carotenoids could be expedited in plant tissue from days to minutes using our newly developed immediate stress technique, then we would be able to observe this process more closely. One of the important advantages of Raman mapping is the ability to monitor distributions of molecules in real-time (Kann et al., 2015). In addition, we addressed the question of which plastids display carotenoid degradation during an osmotic stress such as drought. Carotenoid contents vary in different types of plastids, for example chlorophyll containing chloroplasts or chlorophyll-less leucoplasts such as etioplasts (Sun et al., 2018). To separately investigate etioplasts and chloroplasts, we grew individual seedlings simultaneously; one in complete darkness and the other under normal light. The plants that grew in darkness did not produce mature chloroplasts and thus could not perform photosynthetic functions (Alberts et al., 2002), whereas the green plants contained photosynthetically active chloroplasts (Figure 2A). To understand the subcellular origin of carotenoids being degraded under osmotic stress, we compared the responses of leaf disks from the chlorophyll-less plants that grew under complete darkness that had few if any mature chloroplasts to those that contain normal levels of fully mature chloroplasts. For this, the excised leaf disks were subjected to osmotic stress conditions while taking Raman measurements.

For Raman spectroscopy, a single frequency laser light is used to excite molecules. The excited molecules emit light with new optical frequencies that are downshifted from the incident laser frequency by the amount equal to the molecule vibrational frequencies. This new optical frequency of light (referred to as Stokes radiation) is then measured by a spectrometer. For example, the average Raman spectra of the white and green tissue of live plants are shown in Figure 2B, with different intensities of carotenoid's Raman peaks at 1,157 and 1,007 cm^−1^. Note that normally, carotenoids have the strongest Raman peak at 1,524 cm^−1^, but we couldn't use it here because it overlaps with the anthocyanin Raman peak that also exists in plant tissue (Altangerel et al., 2017b). Since Raman peak intensity directly correlates with the concentration of molecule, we observed that the chlorophyll-less plants had less carotenoids compared to the chlorophyll containing plants in Figure 2B. Indeed, etioplasts are known to accumulate only limited amounts of carotenoids (Lintig et al., 1997; Welsch et al., 2000).

Next, we simulated osmotic stress in the chlorophyll containing (green) and chlorophyll-less (white) leaf disks using our newly developed osmotic stress protocol (Figure 1). The leaf disks from each plant were exposed to 150 mM Mannitol solution and we took Raman XZ and XY maps before and after the stress at specific time points. According to Raman maps in Figure 2C, one can see clearly that carotenoids emitting at the 1,157 cm^−1^ Raman line did not degrade in white plant tissue during the osmotic stress, yet they did in green plant tissue. This selective degradation seen in the green plants demonstrates that carotenoids in chloroplasts preferentially degraded during osmotic stress compared to carotenoids in etioplasts (Figure 2D).

**Figure 3:**
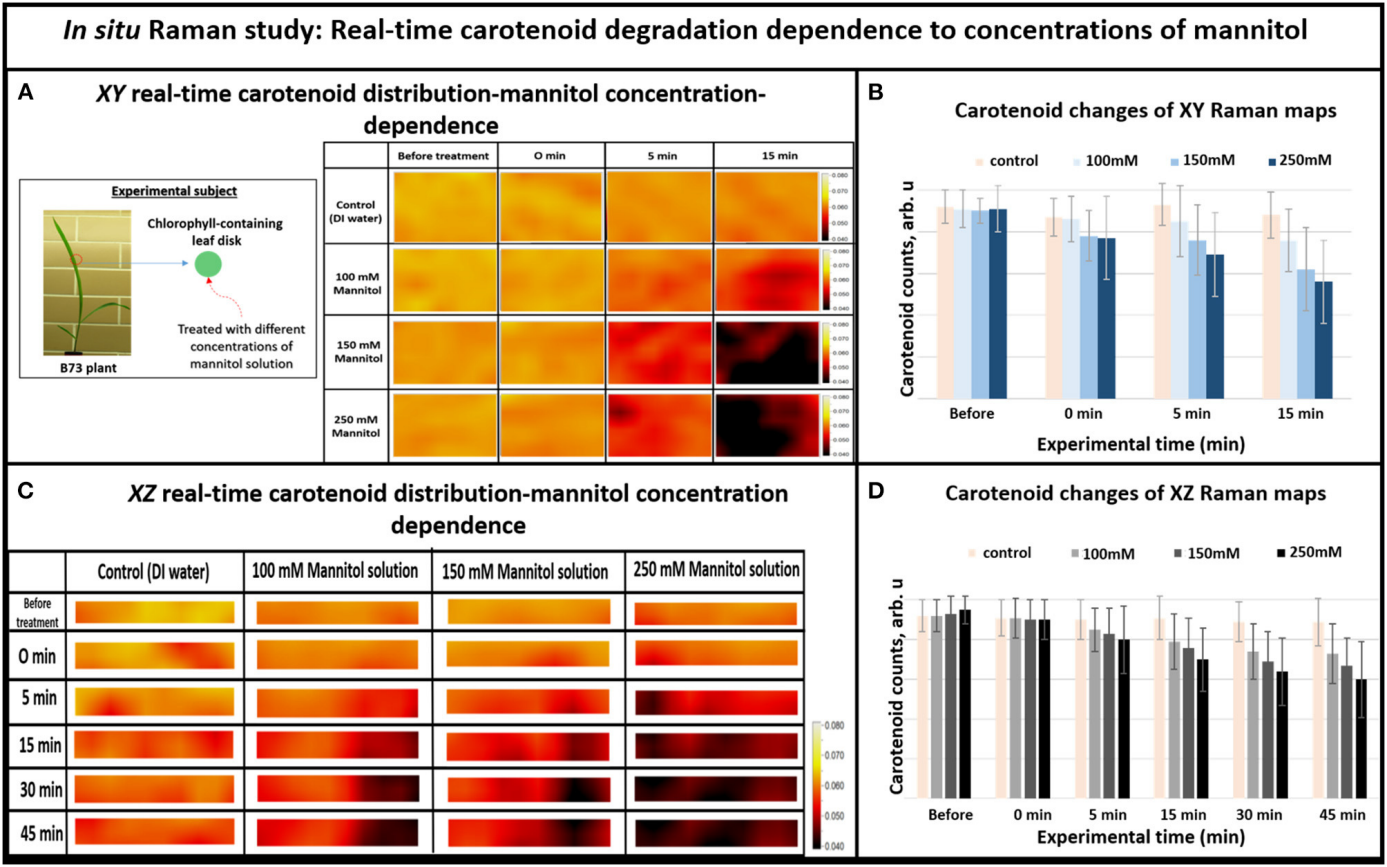
**(A)** XY Real-time carotenoid distribution changes depend on the mannitol concentrations of 0, 100, 150, and 250 mM, before the treatments and 0, 5, and 15 min after the treatments (the green leaf disks-areas of 170 × 140 μm), **(B)** Histogram of carotenoid changes of XY Raman maps. Each bar represents mean +SD (*n* = 80 × 3 × 2), **(C)** XZ Real-time carotenoid distribution changes depend on the mannitol concentrations of 0, 100, 150, and 250 mM, before treatments and 0, 5, 15, 30, and 45 min after the treatments (the green leaf disks where z is depth inside the tissue-areas 170 × 2 μm), **(D)** Histogram of carotenoid changes of XZ Raman maps. Each bar represents mean +SD (*n* = 30 × 3 × 2).

Furthermore, we investigated whether Raman technique can sense shifts of carotenoid degradation rate during different levels of the osmotic stress in the chlorophyll containing leaf disks. In general, higher concentrations of mannitol solution should result in faster dehydration rate, which linearly correlates with faster carotenoid degradation. As shown in Figure 3, the Raman images for carotenoid distributions in plant tissues (intensities at 1,157 cm^−1^ Raman line) show different strengths of stress for different concentrations of mannitol solutions. One can see that carotenoids are distributed evenly before stressing, suggesting that they exist in similar concentrations throughout the plant tissue under normal conditions. After the dehydration solution was introduced into plant tissue, the pattern of carotenoid distribution changed in minutes in response to different concentrations of mannitol solutions. For example, significant carotenoid degradation was seen throughout the mapped area after 5 min exposure to 250 mM mannitol solution, whereas similar level of degradation took 15 min with 100 mM solution.

Overall, when we increased the mannitol concentration from 100 to 250 mM, the Raman XZ (tissue depth) maps (Figure 3C) show that the carotenoid, due to re-distribution or degradation, changed rapidly between 5 and 15 min after the stress. Similarly, the XY Raman images (Figure 3A) show that carotenoid degradation also intensifies in response to treatment with mannitol especially between 5 and 15 min after the stress. These results confirm that this new osmotic stress protocol indeed imitates osmotic stress in tissue as reported in our previous study (Altangerel et al., 2017a), and that Raman mapping is able to accurately sense the degradation rate in carotenoid levels caused by different strengths of osmotic stresses.

### *In vivo* Raman Study: Carotenoid Degradation in Maize Inbreds Due to Drought Stress

**Figure 4:**
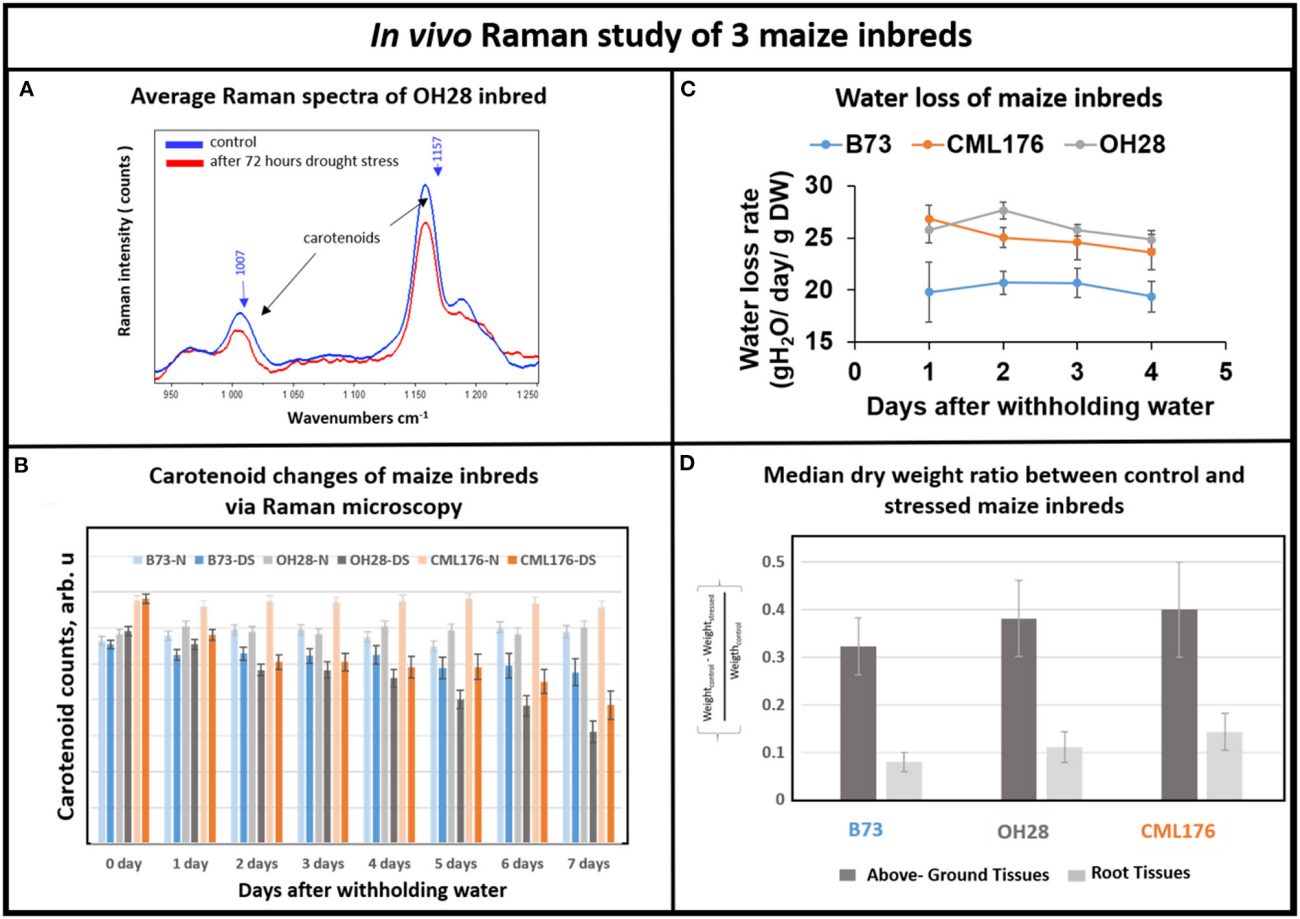
**(A)** Average Raman spectra of OH28 inbred: control vs. 72 h after drought stress. **(B)** Histogram of a change in the carotenoid content of plants at B73-N (control), B73-DS (drought stressed) CML176-N (control), CML176-DS (drought stressed), OH28-N (control) and OH28-DS (drought stressed). Each bar represents the mean +SE (*n* = 3x5). **(C)** Transpirational water loss rate of the inbreds. **(D)** Histogram of dry weight ratio between the control and the stressed plants at B73, CML176, and OH28. The higher the value the more weight of tissue is lost and the more sensitive the plants are to drought stress.

Next, we investigated whether Raman technique can distinguish drought tolerance levels in response to withholding water for seven days in genetically diverse maize inbred lines including B73, CML176, and OH28 by comparing their carotenoid degradation rates. In Figure 4A, we compared the average Raman spectra of OH28 inbred line before and after 72 h of stress, focusing on the intensities of the carotenoid Raman peaks at 1,157 and 1,007 cm^−1^. The observed decrease allows us to conclude that carotenoids are degraded during these 2 days. Figure 4B shows the mean amplitudes of the carotenoid peak at 1,157 cm^−1^ of each genotype from the onset of the experiment to day 7. The rate of carotenoid degradation serves as a proxy how plants respond to drought stress. The faster the carotenoids degrade under drought stress, the less carotenoids left in the plant tissue to scavenge damaging ROS resulting in increased sensitivity to drought stress. Therefore, the germplasm that exhibits greater degradation rate will likely be less drought tolerant. According to our Raman measurements (Figure 4B), carotenoids were degraded less in B73 and more in CML176. In particular, the relative carotenoid degradation rates (from day 0 to day 7) were 22, 20, and 6%, respectively for CML176, OH28, and B73 inbreds. At the 0.05 level, the population variances showed no significant difference between 3 replicate experiments. These results, therefore, suggest that B73 is the most drought-tolerant maize inbred and CML176 is the least tolerant.

To measure relative drought tolerance levels in these inbred lines directly, we measured how much water was lost transpirationally in response to withholding water (Figure 4C). The results show that CML176 and OH28 plants had the highest transpirational water loss rate, indicating that they are more drought sensitive. In contrast, B73 displayed the lowest level of water loss through transpiration and therefore is the most drought tolerant among these inbreds. The normalized dry weight loss for control vs. stressed plants (Figure 4D) agrees with the water loss data. In particular, CML176 had the biggest differences in weight between the control and stressed plants for both above ground and root tissues during the week-long water deprivation. In contrast, B73 had the least differences between control and stressed plants for both tissues. Overall, these results agreed with our inbred line behavior as expected from the carotenoid degradation rates determined by Raman measurements.

### *In vivo* Raman Study: Carotenoid Degradation in Maize Near Isogenic Genotypes Due to Drought Stress

**Figure 5:**
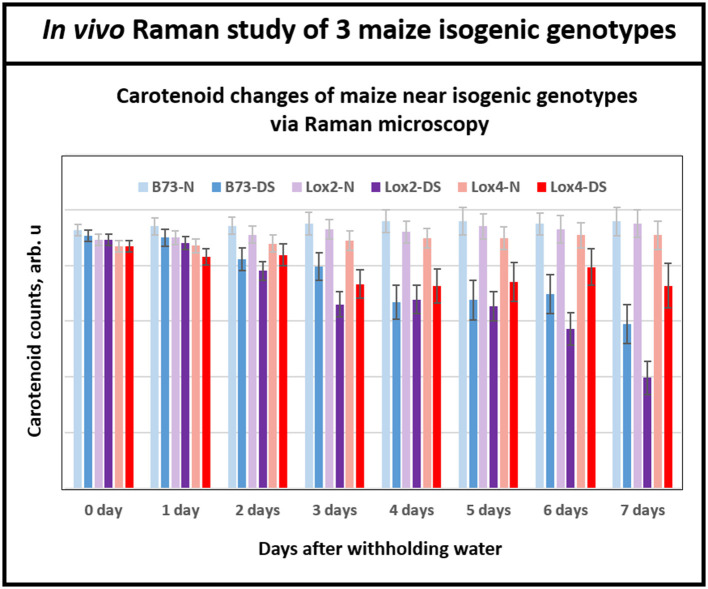
Histogram of a change in the carotenoid content of plants in B73 and near-isogenic lox 2 and lox 4 mutants. While at the beginning of withholding water, all three genotypes showed similar levels of carotenoids, by day 6 and 7 all three genotypes displayed clear separations of carotenoid level between control and drought stressed plants. Each bar represents the mean +SE (*n* = 3 × 5).

Having demonstrated Raman microscopic technique's ability to distinguish the levels of drought tolerance in the genetically diverse maize genotypes, we shifted the focus to using this technique in the analyses of maize genotypes that are near-isogenic to each other at the genome level but display contrasting drought tolerance responses. For this, we chose B73 inbred and two of its near-isogenic lipoxygenase mutants, *lox2* and *lox4*, that were generated by backcrossing 7 times to recurrent parent B73 inbred line, which resulted in their genome being more than 99.2% identical to the parental B73 inbred line. Transpirational water loss experiments with these three near-isogenic lines (NILs) showed that *lox2* is the most drought sensitive line with the highest transpirational water loss compared to B73, and was unable to recover from a prolonged drought stress period after re-watering (Huang, 2017). In sharp contrast, *lox4* is the most drought tolerant genotype displaying lower water loss through transpiration and better survival after 14 days of withholding water followed by re-watering (Huang, 2017). Figure 5 shows that relative average carotenoid degradation rates (from day 0 to day 7) were 10, 6, and 3% for the *lox2, B73* and *lox4* genotypes, respectively. These Raman microscopy results mirror the transpirational water loss experimental results in Huang (2017) and confirm that *lox4* is the most drought-tolerant and *lox2* is the most drought-sensitive line among these three genotypes.

It is important to note that Raman spectra for the near isogenic genotypes in Figure 5 start at nearly the same signal strength for carotenoid levels at the beginning of the experiment, in contrast to Figure 4B for the inbreds. This is because the near-isogenic lines, B73, *lox2* and *lox4*, have almost identical genome sequences and presumably contain near-identical levels of carotenoids, whereas the genetically diverse inbreds (B73, CML 176, and OH28) are quite different. Significantly, Raman technique provides the tool to clearly separate the responses of the near-isogenic genotypes after a week-long drought by monitoring degradation of carotenoids. The existing techniques for measuring carotenoids in plants such as reflectance and fluorescence spectroscopies offer low cost but their sensing time is not as fast as Raman. In our previous study with *Coleus* plants (Altangerel et al., 2017a), we collected reflectance and fluorescence spectra from the experimental plants in addition to Raman measurements. The average reflectance, fluorescence and Raman spectra of the experimental plants on the onset of the experiment and 60 h after the drought stress are shown in the Supplementary Figure 1. The Raman spectra showed significant changes, whereas no changes were observed in the reflectance and fluorescence spectra.

## Conclusions

Carotenoid degradation in plant tissues is one of the plant's first defense against ROS. Since we are detecting these carotenoid molecules in response to drought stress, rather than detecting other physiological changes, we were able to rapidly correlate the dynamics of these biomolecules to the plant tolerance levels. The high accuracy of Raman technique shows that the degradation rate of carotenoids is a reliable indicator of the drought tolerance levels in the genotypes of maize with different levels of drought tolerance.

Raman mapping technique enabled us to observe the real-time dynamics of the changes of carotenoid content in the plant leaf tissue during osmotic stress. In this *in-vitro* study, we found that the distribution of carotenoids in dark-grown (white) tissue didn't change during osmotic stress, and that chlorophyll-less plastids had lower content of carotenoids compared to chrophyll-containing chloroplasts found in the green tissue. The results also showed that only carotenoids in the chloroplast were degraded during osmotic stress.

Most importantly, we demonstrated that Raman technique is a robust, non-invasive, and biochemically selective phenotyping technique that could distinguish drought-tolerance levels not only in genetically diverse genotypes (maize inbred lines) but also in near-isogenic genotypes that contrast in their drought tolerance levels. The robustness of this technique is that it can be performed with two-week old seedlings and requires only a weeklong withholding of water. Climate change increases the odds of a short period of drought stress to happen under natural field conditions in most parts of the world. Thus, using this technique can expedite breeding processes carried out in many areas that do not otherwise provide reliable drought stress conditions. Moreover, this *in vivo* phenotyping technique offers a rapid and accurate method to select drought tolerant genotypes.

In summary, the versatility of Raman techniques allows us to monitor degradation of carotenoids within days in live plants and within minutes in plant leaf disks during drought stress. The robustness and accuracy of this technique offers a great opportunity for future development of high-throughput screening for plant phenotyping. Because hand-held Raman devices are commercially available, this technique has the capability to become mobile and automated, allowing for improved-precision agricultural applications for both breeders and commercial producers.

## Data Availability Statement

The original contributions presented in the study are included in the article/Supplementary Material, further inquiries can be directed to the corresponding author.

## Author Contributions

NA: investigation, data curation, and methodology. P-CH: investigation and methodology. MS, MK, and PH: methodology, funding acquisition, and supervision. All authors contributed to the article and approved the submitted version.

## Conflict of Interest

The authors declare that the research was conducted in the absence of any commercial or financial relationships that could be construed as a potential conflict of interest.
