# Supplementary material for: Raman Spectroscopy as a Robust New Tool for Rapid and Accurate Evaluation of Drought Tolerance Levels in Both Genetically Diverse and Near-Isogenic Maize Lines
Source: Front Plant Sci. 2021 Jul 12;12:621711. doi: 10.3389/fpls.2021.621711 (PMC8311160; doi:10.3389/fpls.2021.621711)
Supplement: Supplementary file 1 [file Data_Sheet_1.PDF]

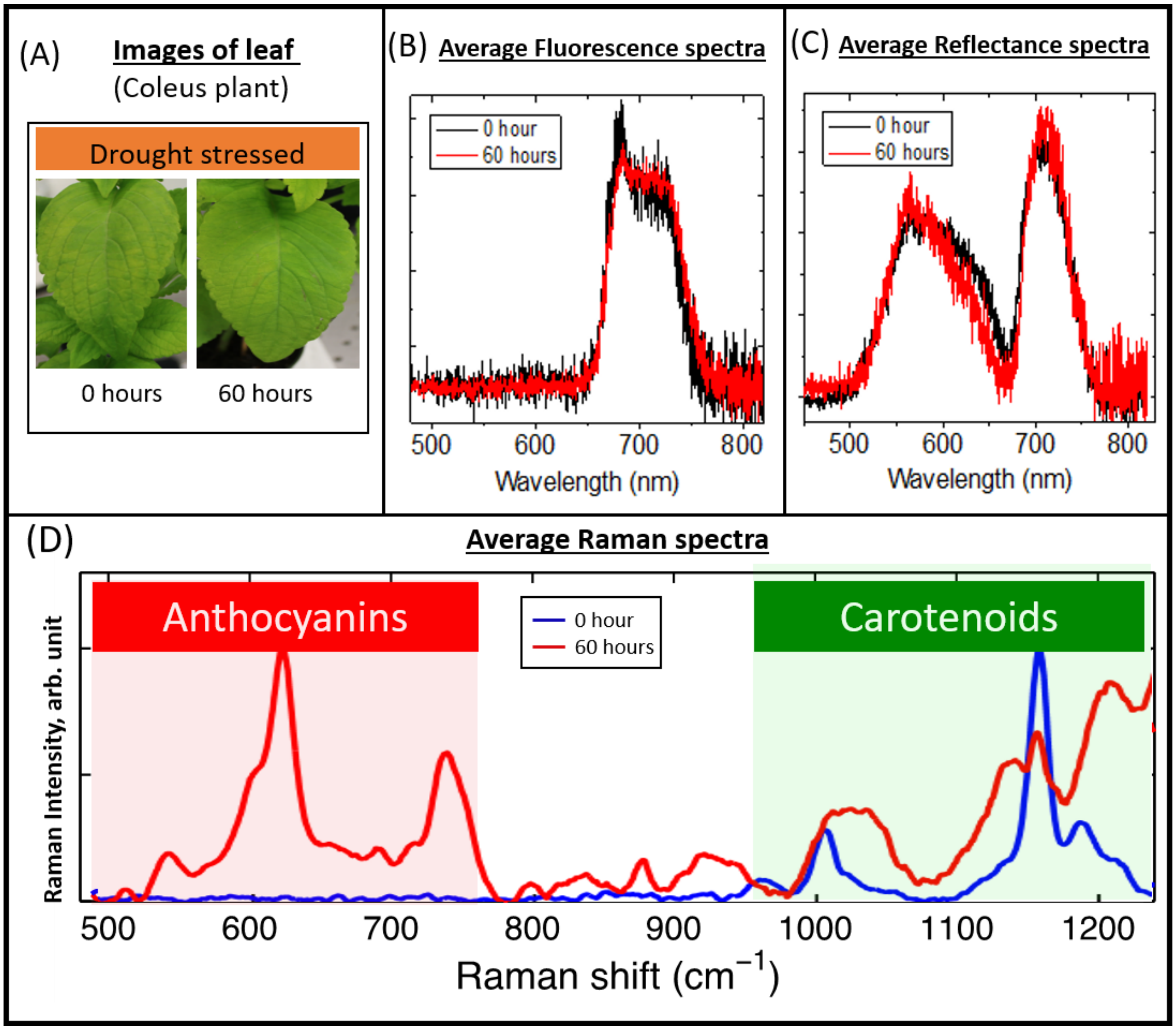

Figure S1: A) Images of coleus leaves for (left) unstressed and (right) stressed, B) Average fluorescence spectra of unstressed plants (black curve) and stressed plants at 60 hours after stress (red curve) (n=2x2x4), C) Average reflectance spectra of unstressed plants (black curve) and stressed plants at 60 hours after stress (red curve) (n=2x2x4), D) Average Raman spectra of unstressed plants (black curve) and stressed plants at 60 hours after stress (red curve) (n=20x4x4).
